# Supplementary material for: Angiotensin-converting enzyme 2 (ACE2) expression increases with age in patients requiring mechanical ventilation
Source: PLoS One. 2021 Feb 16;16(2):e0247060. doi: 10.1371/journal.pone.0247060 (PMC7886150; doi:10.1371/journal.pone.0247060)
Supplement: S1 File — (DOCX) [file pone.0247060.s001.docx]

**Supplement for Angiotensin-converting enzyme 2 (ACE2) expression increases with age in patients requiring mechanical ventilation.**

Steven Andrew Baker, Shirley Kwok, Gerald J. Berry, Thomas J. Montine

This document contains: Page

S Methods. 1

S1 Fig. H&E stained images of tissue from main text figures. 3

S2 Fig. Image processing pipeline for ACE2 and cellular quantification. 4

S3 Fig. Incorporation of sample ischemic time doesn’t alter the effect of age on *ACE2* expression. 5

S1 Table. The relationship of age to tissue *ACE2* expression across the human body. 6

S4 Fig. IHC for ACE2 reveals expected results in normal human kidney and small intestine. 7

S5 Fig. Mouse scRNAseq indicating which lung cell types express detectable *Ace2*. 8

S2 Table. Patient information for lung cases used in this study. 9

S6 Fig. The abundance of alveolar macrophages does not change with age in ventilated patients. 10

S7 Fig. Lung ACE2 staining correlates with age but not other features in ventilated patients. 11

S References. 11

**S Methods.**

**GTEx RNA sequencing analyses***.* Data for bulk tissue RNA expression level in TPM were downloaded from the portal [1] (GTEx_Analysis_2017-06-05_v8_RNASeQCv1.1.9_gene_tpm.gct). Subject data was acquired from (GTEx_Analysis_v8_Annotations_SubjectPhenotypesDS). Donor age, binned by decade, was treated as a continuous variable and sex as a binary variable. The Hardy scale scores are described by the GTEx consortium [2] as representing the length of the terminal phase culminating in death for each donor with a score of 1 representing a violent and fast death lasting <10 minutes, a score of 2 representing a fast death by natural causes lasting 10 minutes – 1 hour, a score of 3 representing an intermediate rate of death lasting 1 hour – 24 hours, a score of 4 representing a slow death with a terminal phase lasting > 24 hours, and a score of 0 representing donors supported by a ventilator preceding death. A small number of donors in the dataset were not given a Hardy score which is indicated here as unknown. In our linear model the Hardy score was treated as a categorical variable with 6 independent levels (0, 1, 2, 3, 4, and unknown). Thus the β coefficients for each level represent the offset relative to the intercept, β_0_, from which *ACE2* expression was regressed on age. A control for tissue ischemic time was performed using data provided in the sample attribute file from the GTEx portal (GTEx_Analysis_v8_Annotations_SampleAttributesDS) [1].

**Immunohistochemistry (IHC) staining**. Patients were grouped into the ventilated and non-ventilated cohorts based on whether they required mechanical ventilation for AHRF during the admission of and prior to sample collection. Each cohort was stained as a batch. 4µm sections were prepared on SuperFrost Plus slides (Fisher Scientific, cat. # 12-550-15), deparaffinized, and hydrated. Antigen retrieval was carried out in citrate buffer pH 6.0 (Vector Laboratories, cat. # H-3300) at 118°C for 10 minutes. Endogenous peroxidase activity was quenched for 10 minutes in 1% H_2_O_2_. Sections were blocked in 2.5% normal horse serum (Vector Laboratories, cat. # 30022) and incubated for 1 hour at room temperature with a polyclonal rabbit antibody directed against the human ACE2 C-terminus, aa 788-805 (Abcam, cat. # ab15348) diluted 1:1000 in DAKO Antibody Diluent (Agilent, cat # S0809). After washing, sections were incubated in ImmPRESS Reagent Anti-Rabbit IgG Peroxidase (Vector Laboratories, cat# MP-7401) for 30 minutes at room temperature. Staining was carried out using DAKO Liquid DAB + Substrate Chromogen System (Agilent cat. # K3468) for 1 minute at room temperature. Tissue was counterstained with hematoxylin prior to dehydration and mounting in ClearMount (StatLab cat. # MMC0126). Antibody validation/tittering was performed on normal human kidney and intestine. After identifying the optimal conditions for staining, a sample of these tissues was included alongside our lung samples as a control.

**Image Quantification***.* The DAB signal for each section was extracted via the IHC Toolbox for ImageJ using the default settings for H-DAB. The integrated densities from all 5 fields were summed for each sample and taken as the total ACE2 signal. These were either compared directly (i.e., normalized by tissue area) or normalized for the total cellularity by quantitating nuclei in the same fields. This was accomplished by extracting the hematoxylin stain using the ImageJ Colour Deconvolution tool utilizing the default vectors for H DAB. An object mask was generated by a custom script (see below) in ImageJ and nuclei counted using the Analyze Particles tool. The total ACE2 signal was divided by the total nuclear count to generate the average ACE2 intensity per cell.

**ImageJ Script for nuclei counting***.*

run("Colour Deconvolution", "vectors=[H DAB] hide");

close();

list = getList("window.titles");

for (i=0; i<list.length; i++){

winame = list[i];

selectWindow(winame);

run("Close");

}

run("Smooth");

run("Smooth");

run("Smooth");

run("Smooth");

setAutoThreshold("Default");

//run("Threshold...");

setThreshold(0, 160);

run("Close");

//setThreshold(0, 160);

setOption("BlackBackground", false);

run("Convert to Mask");

run("Erode");

run("Erode");

run("Fill Holes");

run("Analyze Particles...", "size=100-2500 show=Outlines exclude include summarize");

close();

close();

**Macrophage Quantification***.* Alveolar macrophages were counted by manual pathologist review from the same raw images used for ACE2 quantification and nuclei counting. Total macrophage counts per 5 20x fields of view and total macrophage counts per total number of cells, as measured by the counted nuclei in each image, were plotted using ggplot2 in R.

**Histologic DAD Score***.* H&E stained sections from each patient in the ventilator cohort were scored based on a modification of the published DAD Score [3]. 4 low-power fields were analyzed for 8 features: alveolar edema, interstitial edema, hemorrhage, interstitial inflammation, epithelial damage/sloughing, epithelial regeneration, microatelectasis, and overdistension. A score from 0 – 3 was assigned for each of these characteristics as follows: 0 – normal appearance, 1 – slight effect, 2 – middle effect, and 3 – severe effect. All scores for each sample were then summed together giving an aggregate DAD Score ranging from 0 – 24. Scoring was performed, blinded to patient demographics, by a board certified pathologist with expertise in lung pathology (GJB).

**S1 Fig. H&E stained images of tissue from main text figures.**

Sections were stained from the indicated patients with H&E and representative images collected. The pathological diagnosis for each case is indicated above the image. **A)** The patient presented in Fig 2 showing normal lung parenchyma. **B)** The patients presented in Fig 3 are shown. The left image corresponds to the patient in Fig 3A and reveals an acute lung injury pattern with hyaline membranes, prominent AT2 cells lining edematous septa. The right image corresponds to the patient in Fig 3B and reveals restructured airspaces with fibrotic widening of alveolar septa, sparse interstitial inflammation and scattered intra-alveolar macrophages. **C)** The patients presented in Fig 4 are shown. The left image corresponds to the patient in Fig 4A and reveals normal lung parenchyma. The right image corresponds to the patient in Fig 4B and reveals normal lung parenchyma. **D)** The patients presented in Fig 5 are shown. The left image corresponds to the patient in Fig 5A and reveals acute lung injury with hyaline membranes. The right image corresponds to the patient in Fig 5B and reveals prominent AT2 cells superimposed on widened fibrotic septa containing chronic inflammatory cell infiltrates. Scale bars indicate 200µm.

**
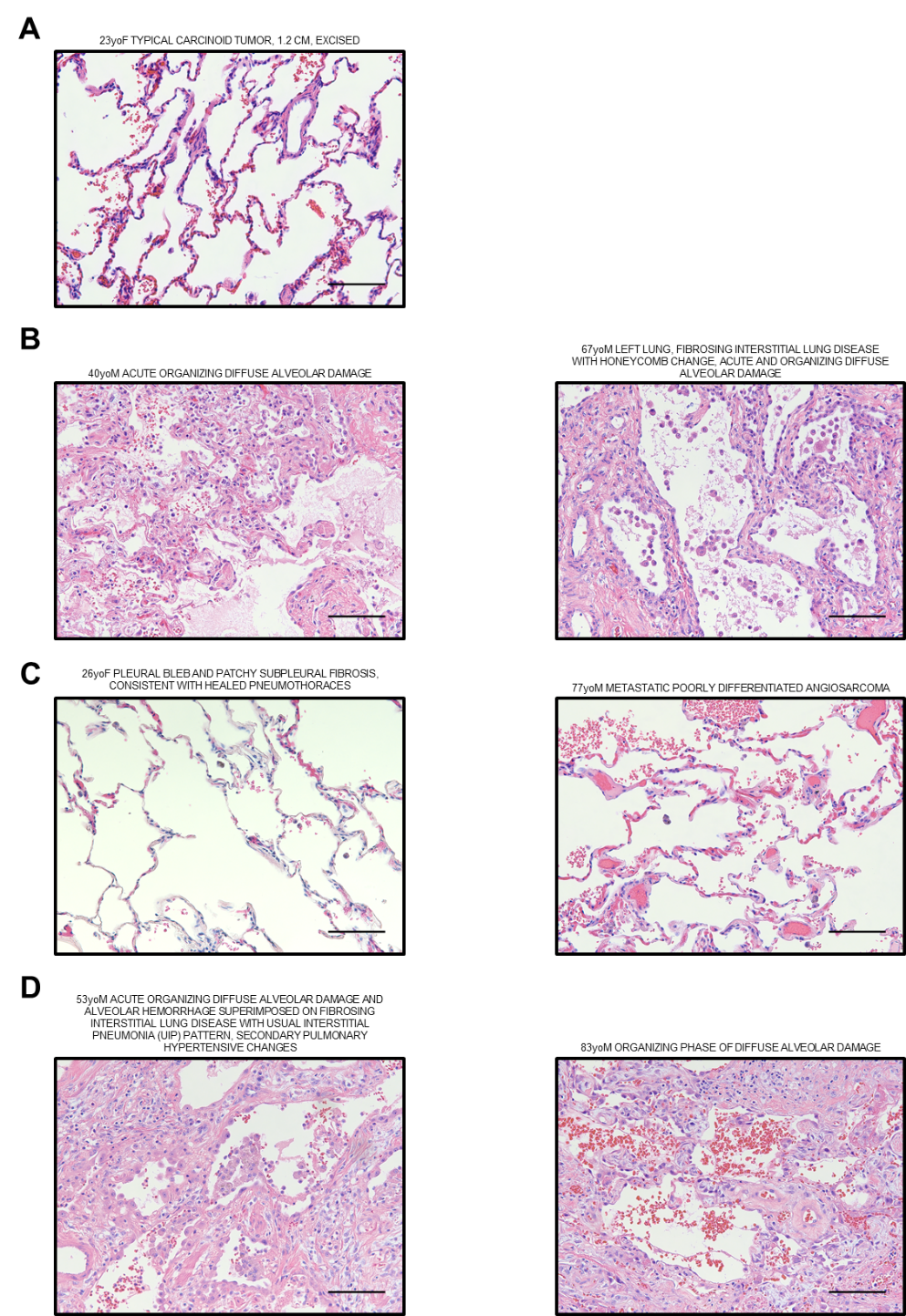
**

**S2 Fig. Image processing pipeline for ACE2 and cellular quantification.**

**A)** Sections used for quantitative comparison were stained in a single batch and imaged under identical conditions without knowledge of patients’ ages. Raw images were processed through an identical pipeline in ImageJ starting with DAB stain extraction using the IHC Toolbox. The histogram was computed for pixel intensity and the integrated density was measured by multiplying the number of pixels by each intensity level. After summing for all 5 20x fields this was taken as the total ACE2 signal. The images shown are taken from the patients presented in Figs 3A and 3B. They reveal the age-related increase in ACE2 seen in the 67yoM on the left compared to the 40yoM on the right. **B)** Cellular counting was performed as described in the **S Methods**. A 20x field is shown above and a zoomed-in portion of the same field is shown at each stage in the pipeline below.


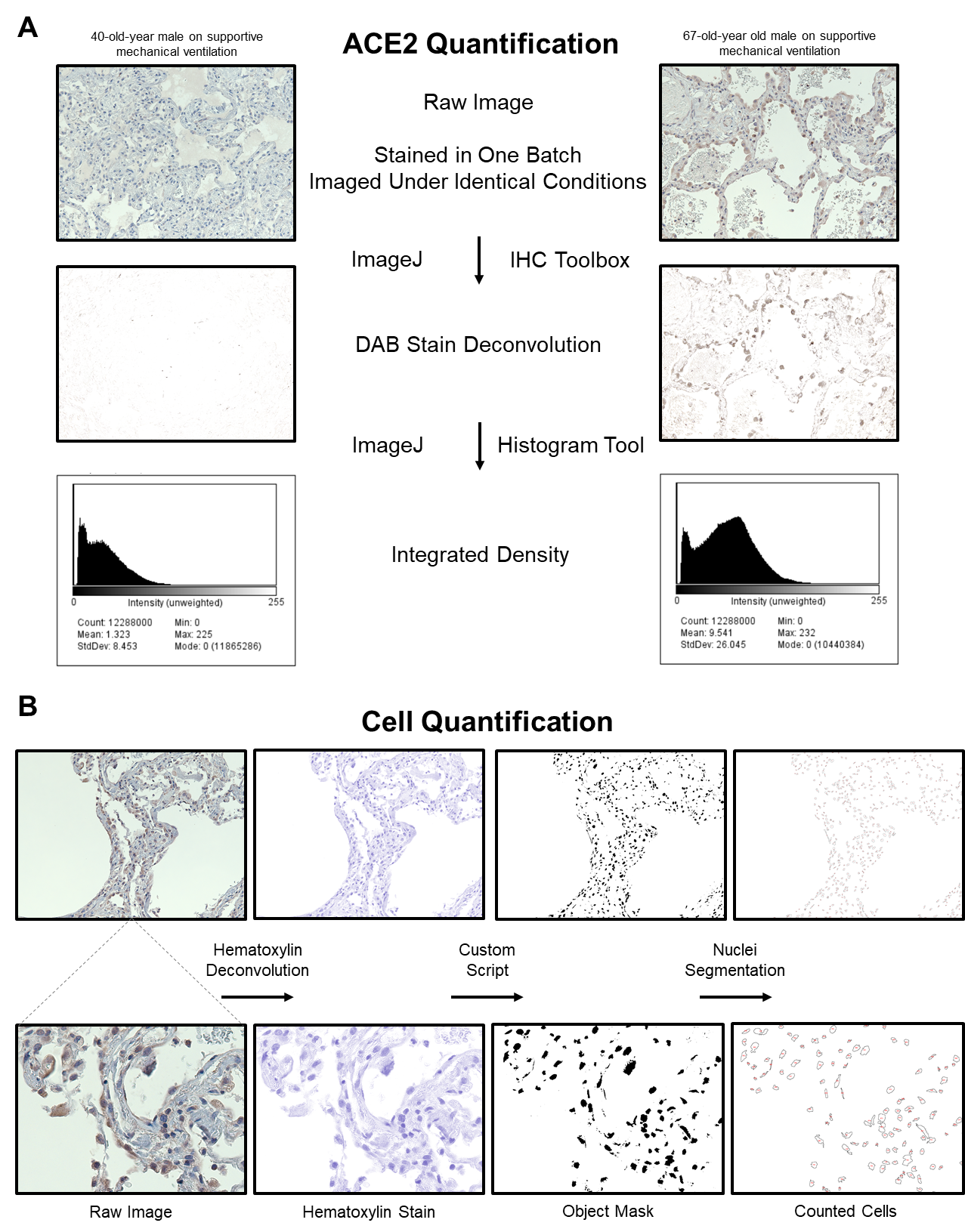


**S3 Fig. Incorporation of sample ischemic time doesn’t alter the effect of age on *ACE2* expression.**

*ACE2* expression in lung stratified by the Hardy scale. Within each Hardy scale group the data are sub-stratified by age. A linear model fit to these data is inset indicating the estimated coefficient for age (β_1_) and for ischemic time (β_2_) as well as their significance. (n = 26 for a score of 1 representing a violent and fast death lasting <10 minutes, n = 156 for a score of 2 representing a fast death by natural causes lasting 10 minutes – 1 hour, n = 31 for a score of 3 representing an intermediate rate of death lasting 1 hour – 24 hours, n = 64 for a score of 4 representing a slow death with a terminal phase lasting > 24 hours, n = 299 for a score of 0 representing donors supported by a ventilator preceding death, n = 2 with an unknown score). Each point represents a sample from a unique individual. The size of the point indicates the length of ischemic time passed between the death of the donor and sample stabilization scaled as per the legend. Box plots indicate quartiles.


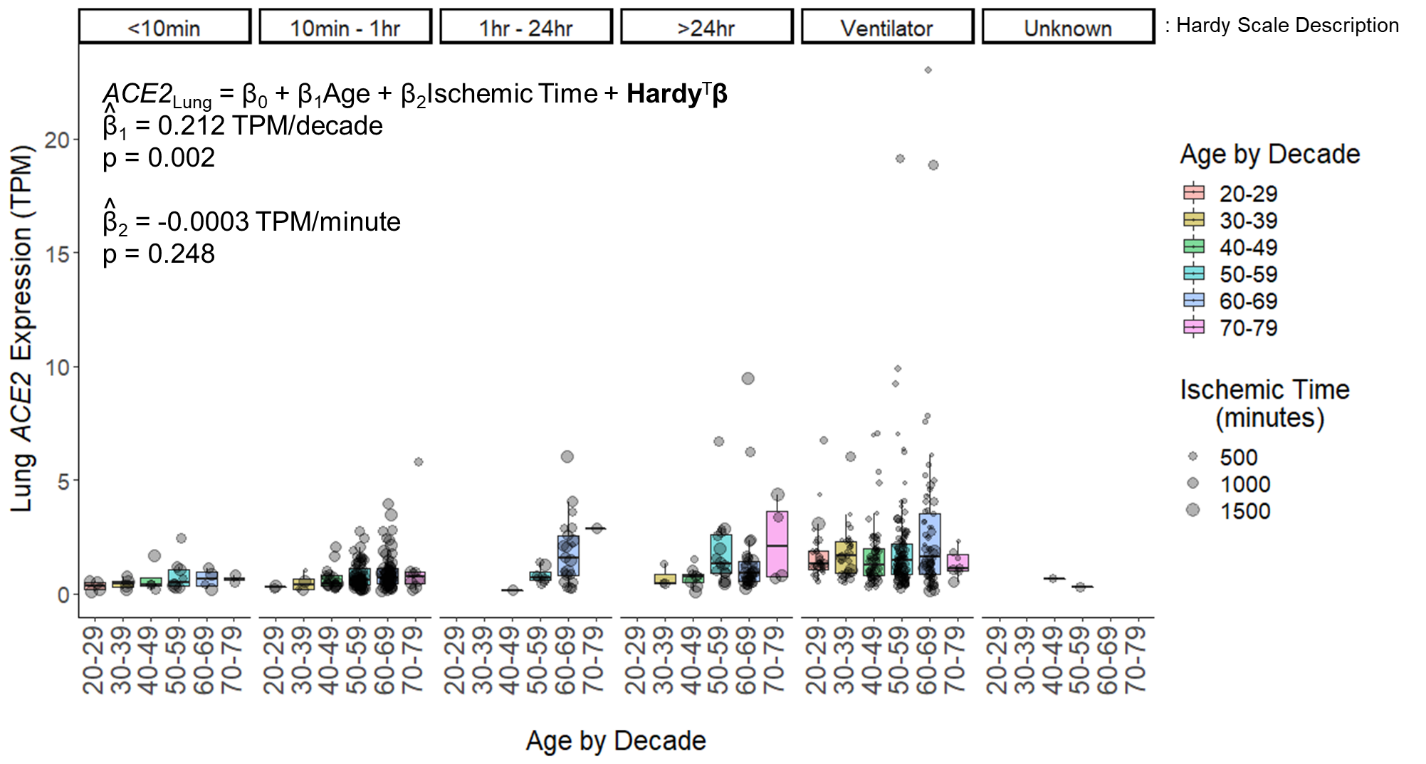


**S1 Table. The relationship of age to tissue *ACE2* expression across the human body.**

A linear model was fit for each tissue: *ACE2* = β_0_ + β_1_Age + **Hardy**^T^**β**; predicting *ACE2* expression with age and Hardy score as covariates for each donor contributing a sample. The coefficient for age and its significance is indicated for each tissue type in the GTEx dataset.

| **Positive Coefficients for Age** |  |  |  | **Negative Coefficients for Age** |  |  |
| --- | --- | --- | --- | --- | --- | --- |
| Tissue | Coefficient | p value |  | Tissue | Coefficient | p value |
| Lung | 0.208 | 0.003 |  | Nerve - Tibial | -0.205 | 0.0003 |
| Artery - Tibial | 0.048 | 0.013 |  | Whole Blood | -0.003 | 0.06 |
| Adrenal Gland | 0.086 | 0.02 |  | Cervix - Ectocervix | -0.605 | 0.069 |
| Liver | 0.152 | 0.021 |  | Minor Salivary Gland | -0.189 | 0.07 |
| Esophagus - Gastroesophageal Junction | 0.2 | 0.028 |  | Bladder | -0.252 | 0.08 |
| Esophagus - Muscularis | 0.209 | 0.033 |  | Colon - Transverse | -0.394 | 0.252 |
| Uterus | 0.187 | 0.041 |  | Pancreas | -0.069 | 0.336 |
| Muscle - Skeletal | 0.056 | 0.042 |  | Cervix - Endocervix | -0.072 | 0.384 |
| Fallopian Tube | 2.696 | 0.124 |  | Testis | -0.664 | 0.44 |
| Thyroid | 0.527 | 0.142 |  | Artery - Aorta | -0.086 | 0.487 |
| Ovary | 1.063 | 0.142 |  | Brain - Cerebellum | -0.003 | 0.594 |
| Artery - Coronary | 0.449 | 0.163 |  | Skin - Sun Exposed (Lower leg) | -0.009 | 0.642 |
| Skin - Not Sun Exposed (Suprapubic) | 0.017 | 0.204 |  | Colon - Sigmoid | -0.053 | 0.665 |
| Small Intestine - Terminal Ileum | 4.357 | 0.245 |  | Adipose - Visceral (Omentum) | -0.238 | 0.668 |
| Heart - Atrial Appendage | 0.223 | 0.249 |  | Pituitary | -0.006 | 0.739 |
| Stomach | 0.07 | 0.282 |  | Prostate | -0.028 | 0.74 |
| Brain - Nucleus accumbens (basal ganglia) | 0.012 | 0.322 |  | Kidney - Cortex | -0.259 | 0.8 |
| Brain - Hippocampus | 0.017 | 0.356 |  | Brain - Cerebellar Hemisphere | -0.001 | 0.838 |
| Breast - Mammary Tissue | 0.405 | 0.358 |  | Adipose - Subcutaneous | -0.027 | 0.932 |
| Vagina | 0.124 | 0.359 |  | Heart - Left Ventricle | -0.028 | 0.938 |
| Brain - Hypothalamus | 0.011 | 0.383 |  |  |  |  |
| Brain - Caudate (basal ganglia) | 0.012 | 0.395 |  |  |  |  |
| Brain - Putamen (basal ganglia) | 0.006 | 0.416 |  |  |  |  |
| Brain - Substantia nigra | 0.06 | 0.416 |  |  |  |  |
| Brain - Spinal cord (cervical c-1) | 0.014 | 0.48 |  |  |  |  |
| Cells - EBV-transformed lymphocytes | 0.001 | 0.61 |  |  |  |  |
| Brain - Cortex | 0.004 | 0.633 |  |  |  |  |
| Brain - Anterior cingulate cortex (BA24) | 0.004 | 0.738 |  |  |  |  |
| Cells - Cultured fibroblasts | 0.002 | 0.822 |  |  |  |  |
| Brain - Amygdala | 0.002 | 0.838 |  |  |  |  |
| Spleen | 0.001 | 0.873 |  |  |  |  |
| Brain - Frontal Cortex (BA9) | 0.002 | 0.899 |  |  |  |  |
| Esophagus - Mucosa | 0.009 | 0.899 |  |  |  |  |

**S4 Fig. IHC for ACE2 reveals expected results in normal human kidney and small intestine. A)** IHC staining for ACE2 in normal human kidney reveals strong signal in the cortex using a 1x objective (left, scale bar 3mm), within cortical tubules at low power (middle, scale bar 200µm), and within brush border cells at high power (right, scale bar 50µm) especially along the apical membrane (red arrowhead). **B)** ACE2 staining in normal human small intestine reveals signal within the luminal surface using a 1x objective (left, scale bar 3mm), along the epithelial lining of villi at low power (middle, scale bar 200µm), and in absorptive enterocytes at high power (right, scale bar 50µm) enriched in the apical membrane (red arrowhead).

**
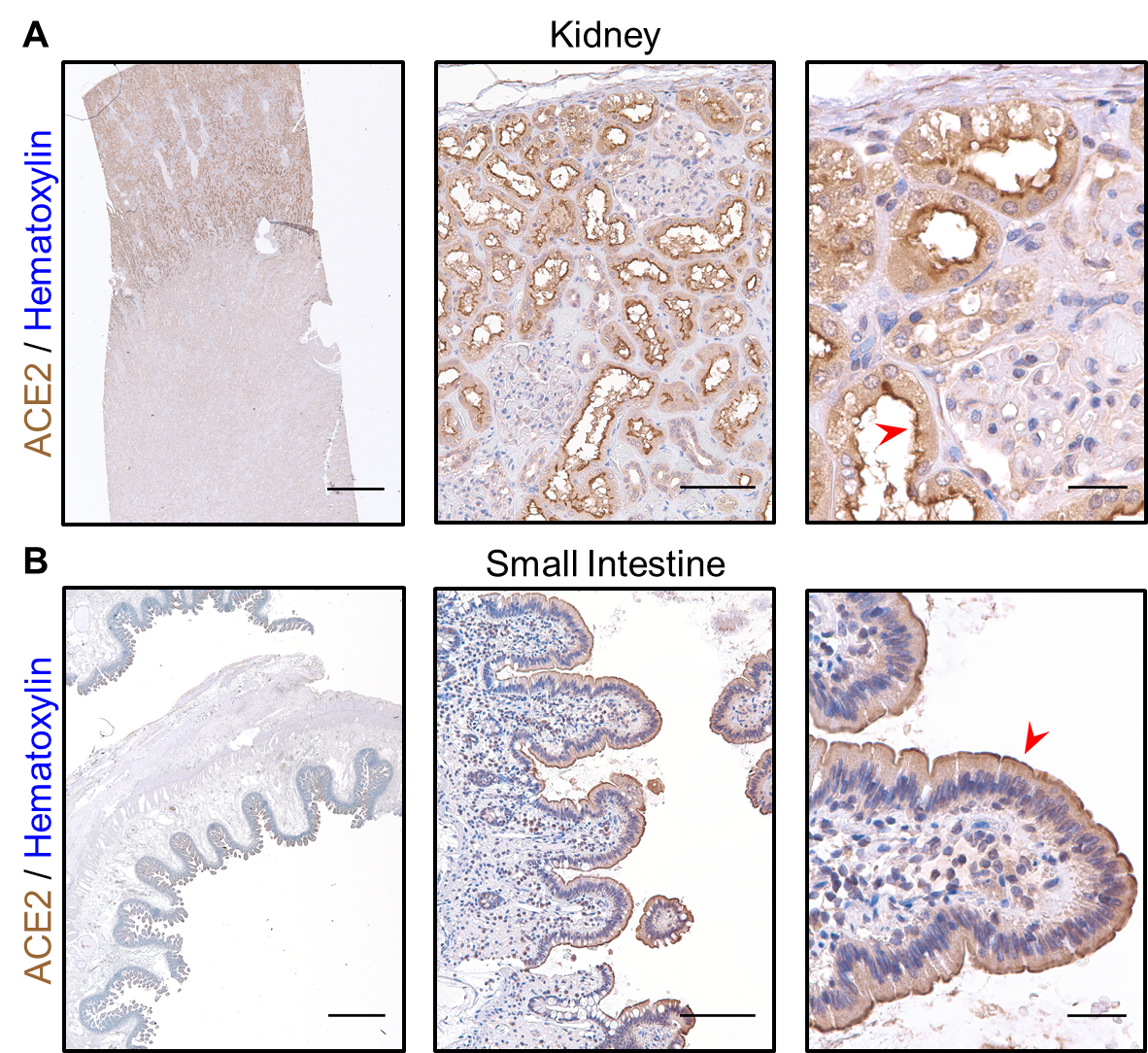
**

**S5 Fig. Mouse scRNAseq indicating which lung cell types express detectable *Ace2*.**

Droplet sequencing data from the Tabula Muris portal were imported into R and the percentages of each cell type in lung with non-zero counts for *Ace2* were plotted. Type II Pneumocytes (AT2 cells) exhibit the highest fraction of detectable *Ace2* expression.


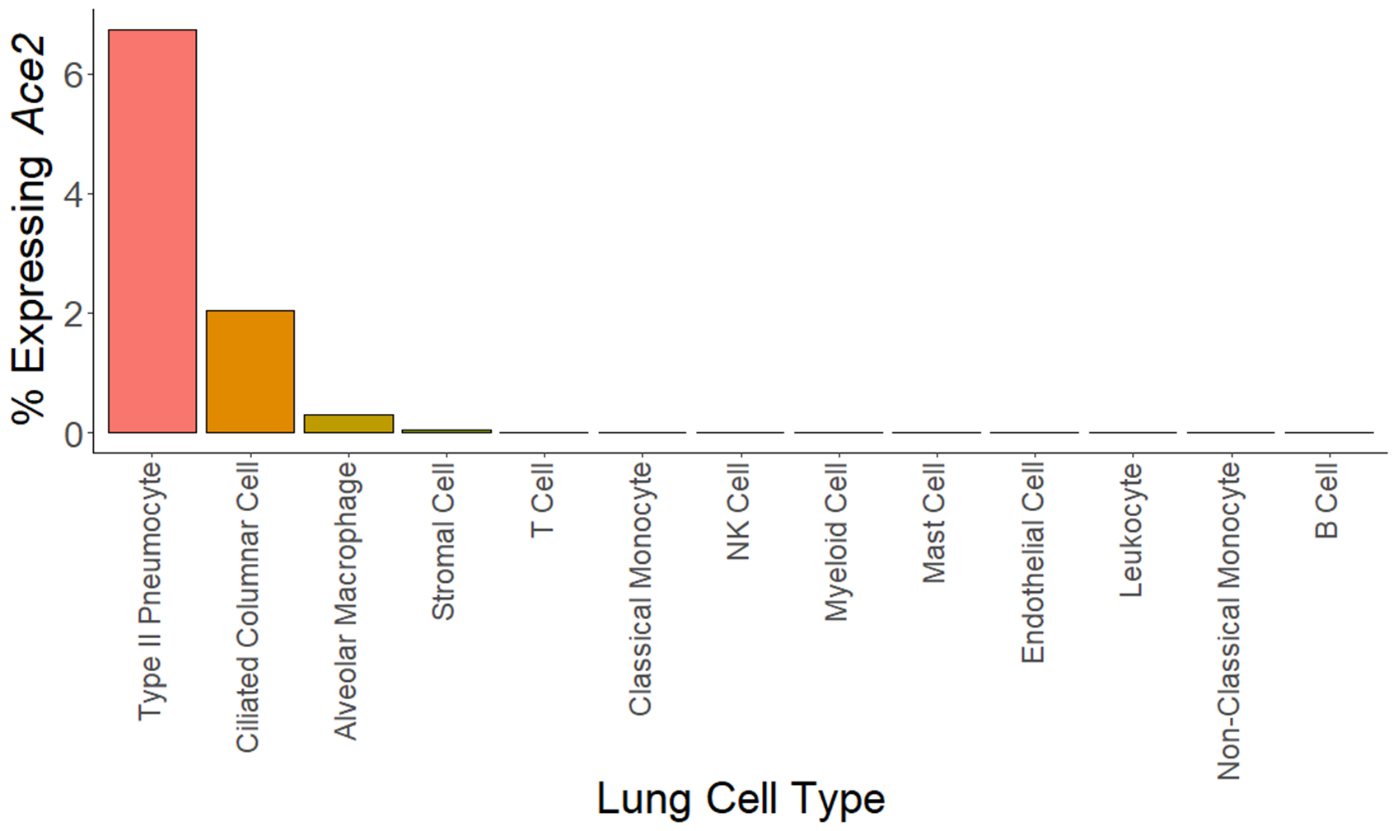


**S2 Table. Patient information for lung cases used in this study.**

The patient age, sex, ventilatory status, prescription information for ACEI/ARB therapy, and pathological diagnosis are given for each sample used. The * denotes the 67yoM sampled for a double lung explant whose tissue was also used as a control for staining/measurement reproducibility as described in the Results section of the main text.

| **Age** | **Sex** | **Supportive Ventilation** | **ACEI/ARB** | **Pathological Diagnosis** | |
| --- | --- | --- | --- | --- | --- |
| 62 | F | Yes | No | DIFFUSE ALVEOLAR DAMAGE | |
| 62 | M | Yes | No | USUAL INTERSTITIAL PNEUMONIA, ORGANIZING PHASE DIFFUSE ALVEOLAR DAMAGE | |
| 53 | M | Yes | No | ACUTE AND ORGANIZING DIFFUSE ALVEOLAR DAMAGE SUPERIMPOSED ON USUAL INTERSTITIAL PNEUMONIA | |
| 51 | M | Yes | No | ORGANIZING PHASE OF DIFFUSE ALVEOLAR DAMAGE | |
| *67 | M | Yes | No | LEFT LUNG, FIBROSING INTERSTITIAL LUNG DISEASE WITH HONEYCOMB CHANGE, ACUTE AND ORGANIZING DIFFUSE ALVEOLAR DAMAGE | |
| *67 | M | Yes | No | RIGHT LUNG, FIBROSING INTERSTITIAL LUNG DISEASE WITH HONEYCOMB CHANGE, ACUTE AND ORGANIZING DIFFUSE ALVEOLAR DAMAGE | |
| 40 | M | Yes | No | ACUTE ORGANIZING DIFFUSE ALVEOLAR DAMAGE | |
| 57 | F | Yes | No | DIFFUSE ALVEOLAR DAMAGE, ORGANIZING PHASE | |
| 83 | M | Yes | Valsartan 160mg bid | ORGANIZING PHASE OF DIFFUSE ALVEOLAR DAMAGE | |
| 47 | M | Yes | No | FIBROSING INTERSTITIAL LUNG DISEASE WITH SUPERIMPOSED ACUTE AND ORGANIZING DIFFUSE ALVEOLAR DAMAGE | |
| 53 | M | Yes | Lisinopril 5mg qd | ACUTE ORGANIZING DIFFUSE ALVEOLAR DAMAGE AND ALVEOLAR HEMORRHAGE SUPERIMPOSED ON FIBROSING INTERSTITIAL LUNG DISEASE WITH USUAL INTERSTITIAL PNEUMONIA (UIP) PATTERN, SECONDARY PULMONARY HYPERTENSIVE CHANGES | |
| 40 | F | Yes | No | ORGANIZING DIFFUSE ALVEOLAR DAMAGE | |
| 14 | M | No | No | BLEB, SUBPLEURAL FIBROSIS | |
| 18 | M | No | No | PLEURAL BLEB AND SUBPLEURAL FIBROSIS | |
| 15 | F | No | No | ALVEOLATED LUNG PAREANCHYMA WITH BLEB FORMATION, SUBPLEURAL FIBROSIS AND REACTIVE CHANGES | |
| 77 | M | No | No | METASTATIC POORLY DIFFERENTIATED ANGIOSARCOMA | |
| 24 | M | No | No | PLEURAL BLEB | |
| 23 | F | No | No | TYPICAL CARCINOID TUMOR, 1.2 CM, EXCISED | |
| 68 | F | No | No | METASTATIC ADENOCARCINOMA CONSISTENT WITH COLORECTAL ORIGIN, 1.4 CM, EXCISED | |
| 18 | M | No | No | PLEURAL BLEB | |
| 26 | F | No | No | PLEURAL BLEB AND PATCHY SUBPLEURAL FIBROSIS, CONSISTENT WITH HEALED PNEUMOTHORACES | |
| 75 | M | No | No | METASTATIC NASOPHARYNGEAL CARCINOMA | |
| 33 | M | No | No | METASTATIC ADENOCARCINOMA, CONSISTENT WITH COLORECTAL ORIGIN, EXCISED | |
| 20 | M | No | No | METASTATIC OSTEOSARCOMA, 1.7 CM, EXCISED | |
| 31 | F | No | No | LUNG PARENCHYMA WITH PLEURAL BLEB FORMATION | |
| 58 | F | No | No | INVOLVED BY LEIOMYOSARCOMA | |
| 70 | F | No | No | TWO FOCI OF METASTATIC COLORECTAL ADENOCARCINOMA, EXCISED | |
| 30 | M | No | No | PLEURAL BLEB; EXCISED | |
| 80 | F | No | No | METASTATIC MALIGNANT PHYLLODES TUMOR, 3.0 CM, EXCISED | |
| 56 | M | No | No | METASTATIC COLORECTAL ADENOCARCINOMA, 1.0 CM; EXCISED | |
| 46 | F | No | No | METASTATIC COLORECTAL ADENOCARCINOMA; 1.1 CM; EXCISED | |
| 49 | F | No | No | HIGH GRADE MALIGNANT NEOPLASM CONSISTENT WITH METASTATIC MALIGNANT PHYLLODES TUMOR; 1.6 CM; EXCISED | |
|  |  |  |  |  |  |
| *Note: this patient had two lung specimens collected at the same time (one form the left lung and one from the right lung with similar findings as indicated in the pathological diagnoses) | | | | | |

**S6 Fig. The abundance of alveolar macrophages does not change with age in ventilated patients.**

**A)** Visual counting of alveolar macrophages was carried out on samples from ventilated patients (n = 12 samples from 11 patients). The total number of alveolar macrophages from 5 low power fields is plotted relative to the patient’s age at the time of specimen collection. A linear fit to the data is indicated by the dashed line with the 95% confidence interval highlighted in grey. **B)** The same specimens quantitated in (A) were normalized by cellularity and the number of alveolar macrophages divided by the total cell count is plotted along with a linear fit to the data and its 95% confidence interval.

**
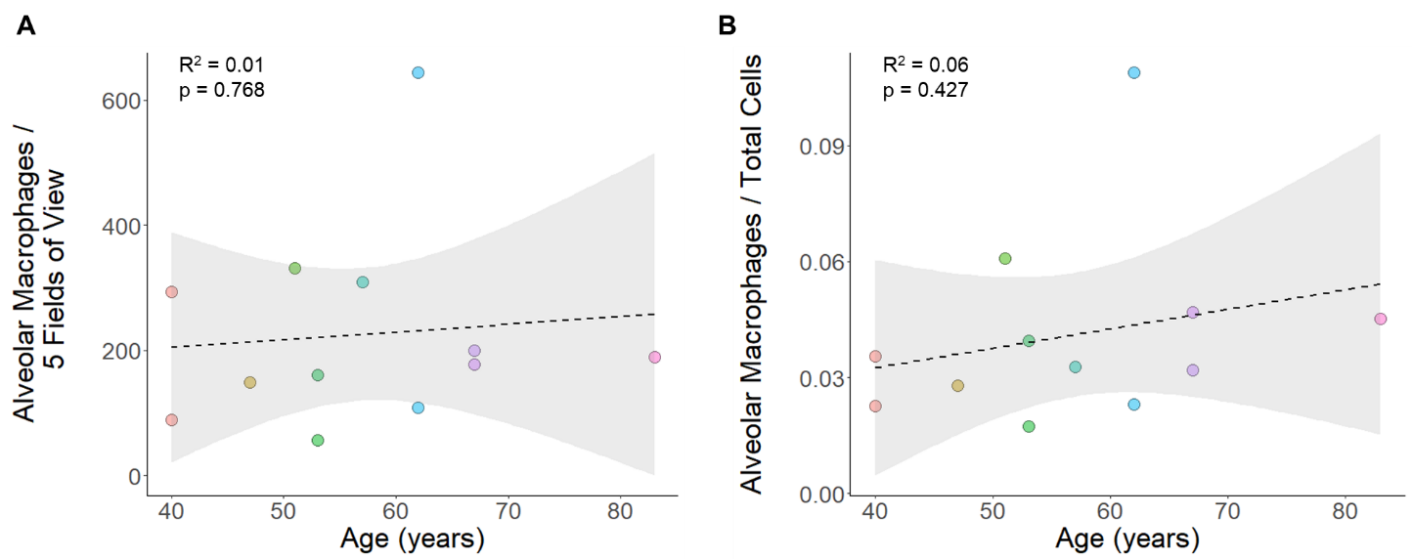
**

**S7 Fig. Lung ACE2 staining correlates with age but not other features in ventilated patients.** Data from chart review for the ventilated cohort are plotted relative to lung ACE2 staining intensity normalized by area (A and C) or cellularity (B and D). n = 12 samples from 11 patients. **A)** Data from Fig 3C (leftmost) are plotted to compare the linear fit of ACE2 staining intensity to (from left to right) sample age, FiO2, PEEP, and the DAD Score. A linear fit to the data is indicated by the dashed line with the 95% confidence interval highlighted in grey. The p value for each model is depicted above the plot, with individual data points colored by patient age. **B)** Similar to (A) except the leftmost plot is from Fig 3D for comparison and data are normalized by cellularity. **C)** For categorical variables a pairwise comparison was made for the presence or absence of (from left to right) ILD, a history of tobacco smoking, or patient sex. The mean +/- 2 SD for each category is shown as a black dot with error bars. The significance of the Wilcoxon rank-sum test between groups is depicted above the plot, with individual data points colored by patient age. **D)** Similar to (C) except normalized by cellularity.

**
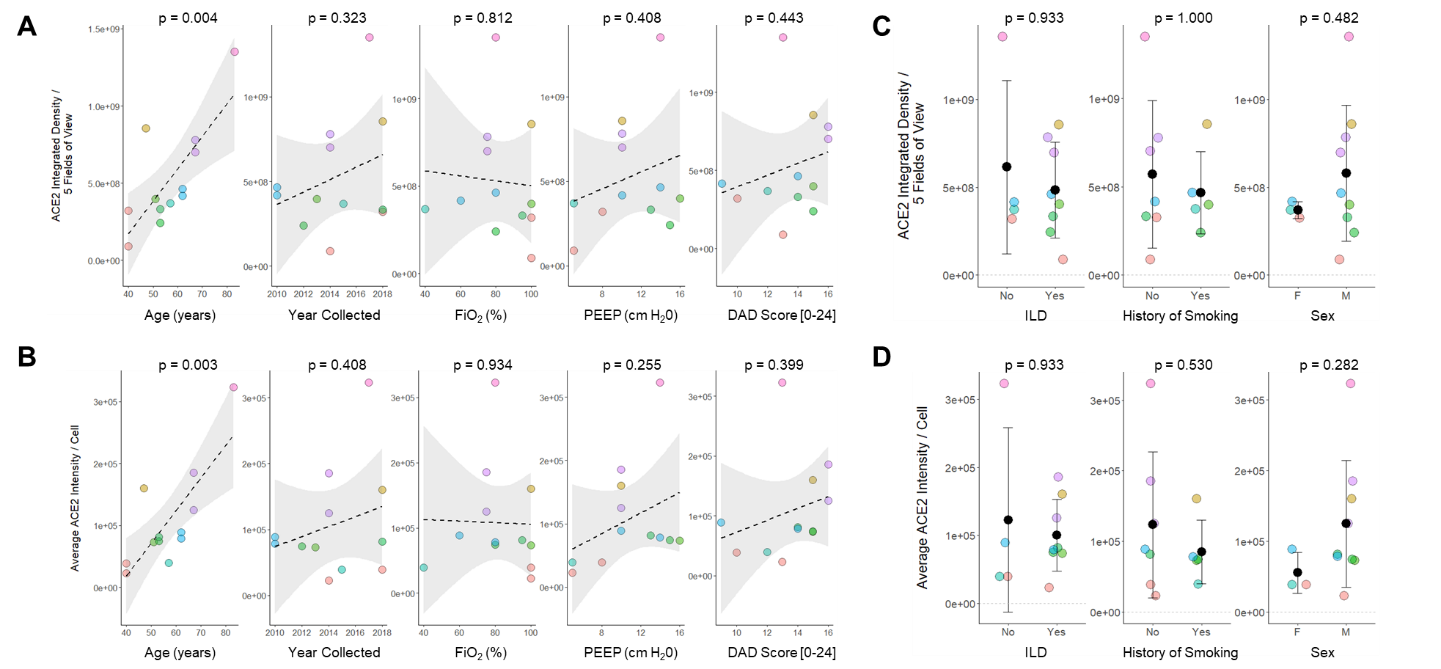
**

**S References.**

1. GTEx Consortium. GTEx Portal. [cited 22 Apr 2020]. Available: https://www.gtexportal.org/home/

2. GTEx Consortium. Hardy scale description. [cited 5 Jun 2020]. Available: https://www.ncbi.nlm.nih.gov/projects/gap/cgi-bin/variable.cgi?study_id=phs000424.v4.p1&phv=169092#:~:text=Death%20classification%20based%20on%20the,phase%20estimated%20at%20%3C%2010%20min.

3. Spieth PM, Knels L, Kasper M, Domingues Quelhas A, Wiedemann B, Lupp A, et al. Effects of vaporized perfluorohexane and partial liquid ventilation on regional distribution of alveolar damage in experimental lung injury. Intensive Care Medicine. 2007;33: 308–314. doi:10.1007/s00134-006-0428-7
